# Organoids of Human Endometrium: A Powerful In Vitro Model for the Endometrium-Embryo Cross-Talk at the Implantation Site

**DOI:** 10.3390/cells9051121

**Published:** 2020-04-30

**Authors:** Alice Luddi, Valentina Pavone, Bianca Semplici, Laura Governini, Mattia Criscuoli, Eugenio Paccagnini, Mariangela Gentile, Giuseppe Morgante, Vincenzo De Leo, Giuseppe Belmonte, Natasa Zarovni, Paola Piomboni

**Affiliations:** 1 Department of Molecular and Developmental Medicine, University of Siena, 53100 Siena, Italy; luddi@unisi.it (A.L.); pavalentina13@gmail.com (V.P.); semplici4@student.unisi.it (B.S.); laura.governini@unisi.it (L.G.); giuseppe.morgante@unisi.it (G.M.); vincenzo.deleo@unisi.it (V.D.L.); giuseppe.belmonte@unisi.it (G.B.); 2 Exosomics, SpA, 53100 Siena, Italy; mcriscuoli@exosomics.eu (M.C.); natasa.zarovni@gmail.com (N.Z.); 3 Department of Life Sciences, University of Siena, 53100 Siena, Italy; eugenio.paccagnini@unisi.it (E.P.); mariangela.gentile@unisi.it (M.G.)

**Keywords:** 3D-cell culture, human endometrium, implantation, Glycodelin A, endometriosis

## Abstract

Embryo implantation has been defined as the “black box” of human reproduction. Most of the knowledge on mechanisms underlining this process derives from animal models, but they cannot always be translated to humans. Therefore, the development of an in vitro/ex vivo model recapitulating as closely and precisely as possible the fundamental functional features of the human endometrial tissue is very much desirable. Here, we have validated endometrial organoids as a suitable 3D-model to studying epithelial endometrial interface for embryo implantation. Transmission and scanning electron microscopy analyses showed that organoids preserve the glandular organization and cell ultrastructural characteristics. They also retain the responsiveness to hormonal treatment specific to the corresponding phase of the menstrual cycle, mimicking the in vivo glandular-like aspect and functions. Noteworthy, organoids mirroring the early secretive phase show the development of pinopodes, large cytoplasmic apical protrusions of the epithelial cells, traditionally considered as reliable key features of the implantation window. Moreover, organoids express glycodelin A (GdA), a cycle-dependent marker of the endometrial receptivity, with its quantitative and qualitative features accounting well for the profile detected in the endometrium in vivo. Accordingly, organoids deriving from the eutopic endometrium of women with endometriosis show a GdA glycosylation pattern significantly different from healthy organoids, confirming our prior data on endometrial tissues. The present results strongly support the idea that organoids may closely recapitulate the molecular and functional characteristics of their cells/tissue of origin.

## 1. Introduction

Embryo implantation is the process by which human embryo orientates towards, attaches to, and finally invades the underlying maternal endometrial tissue. This process requires the establishment of maternal-embryo crosstalk based on a fine-tuning of soluble embryo-derived factors and maternal secreted molecules [1,2,3]. Due to the complexity of the involved mechanisms and the relative lack of information, embryo implantation has been defined as the “black box” within the assisted reproduction procedures. The understanding of the human implantation process is hampered by both ethical issues, not allowing to study this process in vivo, and paucity of reliable, reproducible and flexible in vitro models of human endometrium. To this regard, three-dimensional (3D) human organoids may represent a valuable strategy for reproducing phenotypical and physiological aspects of several tissues [4,5,6,7,8]. Recent studies demonstrated that 3D human organoids may be obtained from the endometrium, opening the way to in vitro study cellular and molecular aspects of this tissue model [9,10,11,12,13]. It is believed that molecules secreted by uterine glands may be important mediators for uterine receptivity, blastocyst implantation, stromal cell decidualization and embryo growth [14,15,16]. In this regard, GdA (also known as placenta protein 14, PP14), is proposed as a strong candidate for a potential biomarker of endometrial receptivity [17]. GdA is one of the four isoforms of glycodelin (Gd), a glycoprotein belonging to the lipocalin superfamily [18]. GdA isoforms are identified according to their differences in glycosylation and sites of production [19]. GdA is produced by the endometrium and, thanks to its distinct glycan chains and characteristic carbohydrate structure, is known to mediate various biological activities in human reproduction and foetal-maternal immunity [20] including immunosuppression, fertilization, implantation and placentation [21,22,23], even if the underlying mechanisms are not completely clarified [22,24,25]. Our research recently demonstrated that GdA expression, and post-translational modifications, change significantly during the menstrual cycle, with glycoforms 4 to 5 being usually well-detectable. Interestingly, a new sixth and more acidic glycoform appears exclusively during the implantation window [26]. This selective expression suggests a crucial role in the endometrial receptivity of this glycoform of GdA that has been already suggested to act as a key regulatory protein during the embryo attachment to the recipient endometrial epithelium [23]. Interestingly, endometrial expression of GdA, as well as its glycoforms composition significantly change during the menstrual cycle in women affected by endometriosis [26]. This hormone-dependent condition that affects up to 5–10% of women in their reproductive age, is characterized by the abnormal growth of endometrial tissue in ectopic sites, outside the endometrial cavity, as well as by a significant reduction in fertility [27].

In this study, we validated the epithelial endometrial organoids as a suitable in vitro model for investigating their responsiveness to hormonal treatments to induce molecular and morphological changes typical of the proliferative and secretory phases of the menstrual cycle. Indeed, we assessed the effectiveness of endometrial organoids in manifesting accurately the molecular mechanisms acting within endometrium at the implantation window, by in-depth analyzing GdA expression in both physiological and pathological conditions, such as endometriosis. Therefore, human endometrial organoids may not only offer extraordinary opportunities for studying embryo-endometrial cross-talk during implantation, but they may be useful in developing and screening of the new prevention and/or treatment strategies for gynaecological pathologies.

## 2. Materials and Methods

### 2.1. Samples Collection

Endometrial specimens used for this study were obtained, with written informed consent from all participants, in accordance with the guidelines in The Declaration of Helsinki 2000 and with the approval of the ethics committee of Siena University, from the Obstetrics and Gynaecology clinic of University Hospital of Siena. Endometrial biopsies have been collected by hysteroscopy from Caucasian women of proven fertility undergoing laparoscopy for tubal sterilization (n = 5), and from women affected by endometriosis undergoing laparoscopic surgery (n = 5). The age of the women ranged from 22 to 39 years and was similar between the two groups (with and without endometriosis: 34.0 ± 3.7 years and 33.1 ± 4.1 years, respectively). BMI ranged between 18 and 25, without a significant difference between the two groups. None of the patients had received any hormonal therapy for at least three months prior to surgery; all participants are not smokers. The stage of the disease was classified according to the American Society for Reproductive Medicine classification and histologically confirmed by pathologists.

### 2.2. Isolation and Culture of Endometrial Glandular Organoids

Endometrial glandular organoids have been isolated from endometrial biopsies as described by Turco and et al. [10], with some modifications. Tissues have been washed in a basic culture medium (Dulbecco’s modified Eagle’s medium, DMEM, plus penicillin 5000 IU/mL and streptomycin 5 mg/mL) and cut into 0.5–1 mm^3^ pieces. Tissue fragments were subjected to enzymatic digestion with collagenase 1 (0,25%)/DNase 1 (0,01 mg/mL) solution in DMEM medium plus 10% FBS (foetal bovine serum). After 1 h in a heated shaker at 37 °C, the cellular/glandular suspension has been passed through 100 µm cell strainers (Corning, Corning, NY, USA) and washed through with DMEM medium (Merck, Kenilworth, NJ, USA). To detach the glandular elements from the membrane, the strainers have been inverted over a petri dish and backwashed by forceful pipetting. They have been collected and centrifuged at 500 *g* for 5 min. Subsequently, the glandular elements have been resuspended in 1–2 mL of DMEM/F12 medium and pelleted by centrifugation. The volume of the pellet has been estimated and added with 20× volume: volume of ice-cold Matrigel Matrix Growth Factor Reduced (BD Biosciences, Franklin Lakes, NJ, USA). We plated 25 µL drops of matrigel/glandular suspension into the 48-well culture plates and placed in the incubator for 15 min. When the matrigel have been solidified, each drop has been overlaid with Expansion Medium (DMEM/F12 containing N2 supplement, B27 supplement minus vitamin A, N-Acetyl-L-cysteine 1.25 mM, L-glutamine 2 mM, recombinant human epidermal growth factor (EGF) 50 ng/mL, recombinant human Noggin 100 ng/mL, recombinant human Respondin-1 500 ng/mL (Peprotech, Rocky Hill, NJ, USA), recombinant human fibroblast grow factor-10 (FGF-10) 100ng/mL, recombinant human hepatocyte grow factor (HGF) 50 ng/mL, ALK-4, -5, -7 inhibitor, A83-01 500 nM, nicotinamide 10 nM, penicillin 5000 IU/mL and streptomycin 5 mg/mL). The organoids were placed in the incubator at 37 °C with 5% CO_2_ and the expansion medium was replaced every 2–3 days.

### 2.3. Human Endometrial Stromal Cells Culture

Human endometrial stromal cells (HESCs) were isolated from healthy endometrial biopsies immediately after collection, accordingly to Luddi et al. [28]. The lower passage number (P0–P4) of cells was used for experiments to avoid changes in phenotype and gene expression. After the hormonal treatment, detailed in the previous paragraph, supernatants and conditioned media were assayed for prolactin using ELISA Kits (Beckman Coulter, San Diego, CA, USA), according to the manufacturer’s instructions. The prolactin concentration range detectable with this reagent set is 0.01–200 ng/mL, and the intra assay and inter assay coefficients of variation were <6% and <10%, respectively.

### 2.4. Hormonal Treatments

Organoids cultures have been exposed to hormonal treatments in order to mimic the endometrial hormonal milieu typical of the proliferative and mid secretory phase of the menstrual cycle. In particular, in order to mimic the proliferative phase, the expansion medium has been supplemented with 10^−8 ^ M E2 (Sigma-Aldrich, St. Louis, MI, USA), while, to mimic the mid secretory phase, the expansion medium has been supplemented with 10^−8^ M E2 + 10^−6^ M P4 (Merck) and 50 mM 8-Bromoadenosine 3′,5′-cyclic monophosphate (cAMP) (Merck). Treated organoids have been cultured for 4 days; after that, organoids have been washed with PBS, detached from the well and centrifuged two times at 600 *g* for 6 min to completely remove matrigel.

### 2.5. Transmission Electron Microscopy (TEM)

For TEM analysis, organoids obtained from healthy and endometriotic women have been resuspended and fixed in cold Karnovsky’s fixative and maintained at 4 °C for 2 h. They were washed with cacodylate buffer 0.1 M pH 7.2 and fixed in 1% buffered osmium tetroxide for 2 h. After they have been washed again with cacodylate buffer, dehydrated in an ascending alcohol series and incubated twice in propylene oxide. They have been infiltrated and embedded in Araldite resin (Merck) that was polymerized at 60 °C for 48 h. Ultrathin sections (600 nm) have been cut from embedded samples on a Reichert Supernova (Leica, Wetzlar, Germany) ultramicrotome, mounted on 200-mesh copper grids and stained with uranyl acetate and lead citrate. Subsequently, they have been observed and photographed with the transmission electron microscope (Tecnai G2 Spirit FEI) operating at an electron accelerating voltage of 120 kV equipped with a Morada (EMSIS) CCD camera. Two independent experiments have been carried out. For each experiment, hundreds of organoids were fixed and included and ultrathin sections at different levels were collected, stained and observed at electron microscopy by two independent operators.

### 2.6. Scanning Electron Microscopy (SEM)

Cultures of human endometrial organoids were fixed for 2 h at 4 °C in cold Karnovsky’s fixative, washed with cacodylate buffer 0.1 M pH 7.2 overnight, post-fixed in 1% buffered osmium tetroxide in veronal acetate buffer for 2 h and washed in cacodylate buffer 0.1 M pH 7.2. Organoids were dehydrated in a graded series of ethanol, placed in tert-butanol and frozen at 0 °C before being dryed by sublimation of the tert-butanol in a vacuum chamber. The samples were cut to expose the internal surface and coated with 20 nm gold/palladium and observed in a Quanta 400 (FEI) scanning electron microscope. Two independent SEM analyses have been performed; for each experiment, hundreds of organoids were fixed and observed at scanning electron microscopy by two independent operators.

### 2.7. RNA Extraction and Droplet Digital PCR (dd-PCR) Analysis

Organoids obtained from healthy women have been lysed with 350 μL of a solution containing RLT (Qiagen, Hilden, Germany) and β-mercaptoethanol. The lysates were centrifuged for 1 min at 13,000 *g* at 4 °C. The pellet was discarded and the supernatants were used for subsequential RNA extraction, conducted by using the Quiacube (Qiagen) automatic extractor, following the manufacturer protocol. At the end of the protocol, RNA extracted from each sample was eluted in a final volume of 30 μL of water. The purity and the concentration of RNA were evaluated by reading on Nano Drop^®^ ND-100 UV-vis Spectrophotometer (Nano Drop Technologies, Waltham, MA, USA).

DNAse treatment (Sigma Aldrich) has been done on RNA samples and gene expression analysis was performed by QX200 droplet digital (dd)-PCR System according to the manufacturer’s instructions (Bio-Rad, Hercules, CA, USA). Each 20 µL pre-amplification ddPCR reaction mixtures were prepared using One-Step RT-ddPCR Advanced kit for probes (Bio-Rad), 5 µL of the sample (≈1 ng) and the specific EvaGreen assays for gene expression (Bio-Rad) (Supplementary Table S1). After droplet generations, samples were amplified with the following cycling conditions: 42 °C for 60 min, 95 °C for 10 min, 39 cycles at 95 °C for 30 sec and 56 °C for 1 min, and 98 °C for 10 min.

Data were analyzed by the QuantaSoft software (BioRad) and results were expressed in terms of fold change using *GAPDH*, *β-Actin*, *HPRT-1*, *B2M* and *TBP* as the housekeeping genes.

### 2.8. Protein Extraction

For protein extraction organoids have been resuspended in RIPA^+++^ buffer (containing 0.05 M Tris; 0.15 M NaCl; 1% Triton X; 0.1% sodium deoxycholate; 0.1% sodium dodecil sulphate (SDS); added with 0.1 M sodium orthovanadate, 0.01 M phenylmethylsulfonyl fluoride (PMSF) and protease inhibitor) for SDS-PAGE and in REID buffer (containing urea 8 M and 4% 3-((3-cholamidopropyl) dimethylammonio)-1-propanesulfonate) for 2D electrophoresis. Each sample underwent three sonication cycles of 10 sec, followed by centrifugation at 13,000 *g* for 10 min. After centrifugation, the supernatant has been collected and the protein concentration of each sample has been evaluated using the Bradford assay.

### 2.9. SDS-PAGE (Sodium Dodecil Sulphate-Polyacrylammide Gel Electrophoresis)

For the SDS-PAGE analysis, 10 μg of total protein lysates were suspended in a sample buffer containing 20% glycerol, 240 mM Tris-HCl, pH 6.8, 8% SDS, 8% β-mercaptoethanol and 0.02% bromophenol blue and then separated by electrophoresis on 10% polyacrylamide gel. The electrophoretic run has been conducted in a buffer containing Tris 0.6%, SDS 0.1%, Glycine 2.88% at 25 mA/gel, using the cell Mini Protean (BioRadMicrosciences).

### 2.10. Two-Dimensional Electrophoresis (2-D Electrophoresis)

2D-electrophoresis was carried out as described: 50 μg of total protein were resuspended in rehydration solution and mixed with 0.2% immobilized pH gradient buffer (IPG, GE Healthcare, Chicago, IL, USA) with a 3–11 nonlinear pH range. Samples were loaded onto ImmobilineDryStrips with immobilized nonlinear pH gradient, ranging from pH 3 to 11 (GE Healthcare). Isoelectric focused strips were equilibrated for 15 min with 50 mM Tris-HCl pH 6.8 containing 30% glycerol, 6 M urea, and 2% SDS and for an additional 5 min in the same solution containing 2.5% iodoacetamide (IAA) and 0.1% bromophenol blue, and then placed on a 10% polyacrylamide gel. 2D-electrophoresis was carried out twice on pooled samples obtained by mixing equal protein amounts (μg of total protein) of 2–3 individual organoid preparations that were extracted in the same lysis buffer. The protein concentration in pooled samples was estimated using the appropriate quantification protocol. Each pool was prepared twice, one for each 2D-electrophoresis.

### 2.11. Western Blotting

For Western blotting analysis, proteins were electroblotted from polyacrylamide gels to nitrocellulose according to Towbin [29] and then blocked in 5% skimmed dry milk in TBS (Tris-buffered saline, 10 mM Tris-HCl, pH 7.5 and 0.15 M NaCl) for 1 hour at room temperature. They were incubated overnight at 4 °C with an anti-GdA antibody direct against the peptidic portion of the glycoprotein (R&D System, Minneapolis, MN, USA) diluted 1:200 in 1% skimmed dry milk/TTBS (TBS containing 0.2% Tween20). After washing, membranes were incubated for 1 h with the appropriated horseradish peroxidase (HRP)-conjugated anti-goat IgG antibody. The reaction was detected using an Immuno-Star HRP Chemiluminescent kit (Bio-Rad). The same nitrocellulose membranes were also incubated with an anti-β-actin antibody followed by the appropriate secondary antibody. The chemiluminescence signals were captured using an XRS instrument ChemiDoc (Bio-Rad). Images were then processed using the Quantity One^®^ 4.5.7 and PDQuestTM 7.4.0 software (Bio-Rad) for bands identification and quantification as pixel/mm^2^.

### 2.12. Immunofluorescence

Formalin-fixed, paraffin-embedded organoids were sectioned at 4-μm thickness, and then fixed in deparaffinized with xylene and dehydrated with ethanol. Antigen retrieval has been obtained by incubation with buffer 10 mM citrate pH 6.0, at a temperature sub-boiling for 20 min. Slides were left to cool for 10 min. Subsequently, they were incubated in a blocker solution containing 5% goat normal serum in PBS/BSA (bovine serum albumin) 1% and then incubated for 1 h at room temperature with the goat anti-GdA polyclonal antibody (1:100; R&D, Abingdon, UK), used according to the manufacturer’s instructions. The specificity of immunostaining was confirmed by both omissions of primary antibody and staining of sections with unrelated antibodies. The slides have been washed in PBS, and the bound antibodies were revealed by incubation with Alexa Fluor 488-labeled rabbit anti-goat IgG antibody (1:100; Thermo Fisher Scientific). After washing in PBS, the slides have been mounted in ProLong antifade with 4′,6-diamidino-2-phenylindole (DAPI) (LifeTechnologies, Carlsbad, CA, USA) to counterstain the nuclei and then they have been observed with a Leica DMB 6000 microscope (Leica). Images have been captured with a CFTR6500 digital camera (Leica).

### 2.13. Statistical Analysis

All experiments described in this work have been reproduced with similar results in independent samples from several patients, by two scientists independently. Statistical analysis was performed with GraphPad Prism version 5.0 (GraphPad Software, San Diego, CA, USA). Standardized skewness and kurtosis values were used to determine the normal distribution of data. Student t-test or Wilcoxon signed-rank test were used when appropriate. Analysis of variance between groups was performed by a one-way analysis of variance test followed by a Bonferroni post-hoc comparison test. Statistical significance was set at *p* < 0.05.

## 3. Results

### 3.1. Established Human Endometrial Epithelial Organoids Respond to Hormones by Recapitulating Morphological and Ultrastructural Features of the Different Phases of the Uterine Cycle

**Figure 1:**
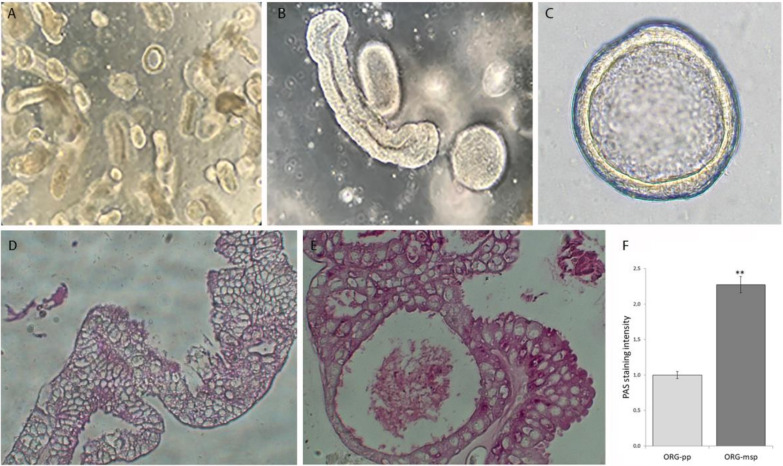
3D-organoid cultures established from human healthy endometrium. (**A**–**C**) Bright field representative images of human organoids from three independent experiments (each from different donors). Scale bar, (**A**): 500 µm; (**B**,**C**): 100 µm. (**D**,**E**) PAS staining for glycogen in organoids treated with E2 (ORG-pp) (**D**) and with E2, P4, cAMP (ORG-msp) (**E**), representative of 3 glandular epithelial organoid preparations. Scale bars, 25 μm. (**F**) Quantification of periodic acid−Schiff (PAS) staining intensity. ** *p* < 0.01.

By using enzymatic digestion of human endometrial biopsies, we isolated glands and epithelial cells that were grown in a defined culture system utilizing matrigel as a scaffold. According to our modified protocol, these epithelial cells are able to self-organize into 3D spherical structures named organoids, that can be expanded for a long time (up to 6 months) without contamination of stromal cells. When observed at low magnification, organoids have a glandular appearance, lined by a columnar epithelium (Figure 1A–C).

The human endometrium is able to respond to ovarian hormones oestrogen (E2) and progesterone (P4) by undergoing specific morphological and molecular changes [30]. Based on this knowledge, we exposed cultured organoids to hormonal treatments mimicking the uterine microenvironment during the menstrual cycle; specifically, we used E2-only treatment to induce the proliferative phase (ORG-pp), whereas E2 + P4 + 8-Bromoadenosine 3′,5′-cyclic monophosphate (cAMP) combined treatment induced the mid-secretory phase (ORG-msp) [10]. When the periodic acid−Schiff (PAS) staining was used to detect the polysaccharide material compatible with glycogen deposition, the intensity of staining was decreased in ORG-pp (Figure 1D) when compared with ORG-msp (Figure 1E), that instead significantly accumulates glycogen and mucus also into the lumen, with a deposition pattern similar to that previously reported in the endometrial tissue in vivo [31].

**Figure 2:**
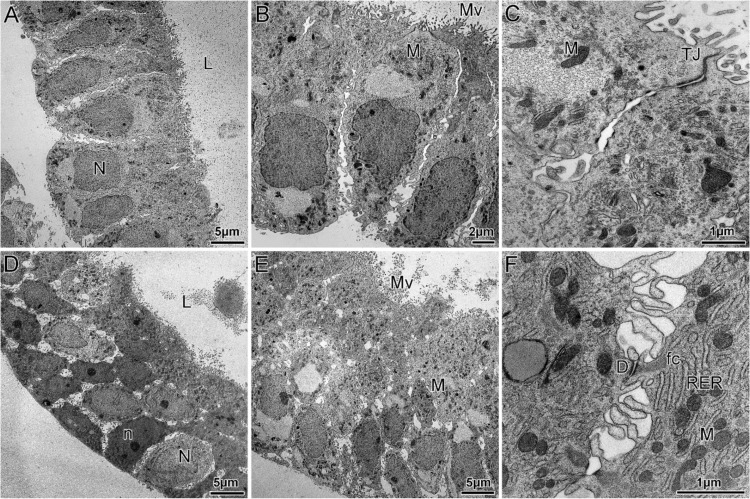
Ultrastructure of epithelial glandular organoids recapitulating the physiological architecture of glands in human healthy endometrium. (**A**–**C**) Representative electron micrographs of ORG-pp treated with E2 to mimic the proliferative phase, showing columnar epithelial cells forming a pseudostratified epithelium. (**D**–**F**). Representative electron micrographs of ORG-msp treated with E2, P4, cAMP to mimic the mid-secretory phase, showing a pluristratified epithelium lining the lumen. L, lumen; N, nucleus; M, mitochondria; Mv, microvilli; TJ, tight junction; D, desmosomes; n, nucleolus; RER, root endoplasmic reticulum; fc, filaments of cytoskeleton.

Transmission electron microscopy (TEM) analysis revealed that ORG-pp (Figure 2A–C) form a pseudostratified epithelium featuring columnar epithelial cells with basally located nuclei and apical microvilli directed toward the lumen, evidencing the apicobasal polarity of these cells (Figure 2A). Ultrastructural characteristics of epithelial cells, such as intact cell-to-cell contacts, may be appreciated; in the upper region of these polarized epithelial cells, we observe regularly distributed electron-dense particles, forming the apical tight junctions, that correspond to the cellular epithelial barrier. These structures separate the lumen from the intercellular clefts between the lateral plasma membranes of two adjacent cells (Figure 2C). TEM analysis also revealed the presence of long mitochondria (Figure 2C), abundant cisternae of rough endoplasmic reticulum and Golgi bodies in the cytoplasm (Figure 2B).

On the other hand, the ORG-msp show a pluristratified epithelium lining the lumen (Figure 2D–F). These epithelial cells are characterized by a number of small round-shaped mitochondria, a higher abundance of Golgi cisternae and, consequently, numerous secretory vesicles highlighting an intense secretory activity at the apical surface. Unlike ORG-pp, in ORG-msp epithelial cells exhibited zonulae adherens with desmosomes to the inner intermediate filament complexes and an increase in the extent of intercellular space with numerous digital expansions of the cell membranes of two adjacent cells, able to connect them as well as to plunge into the intercellular spaces (Figure 2E).

**Figure 3:**
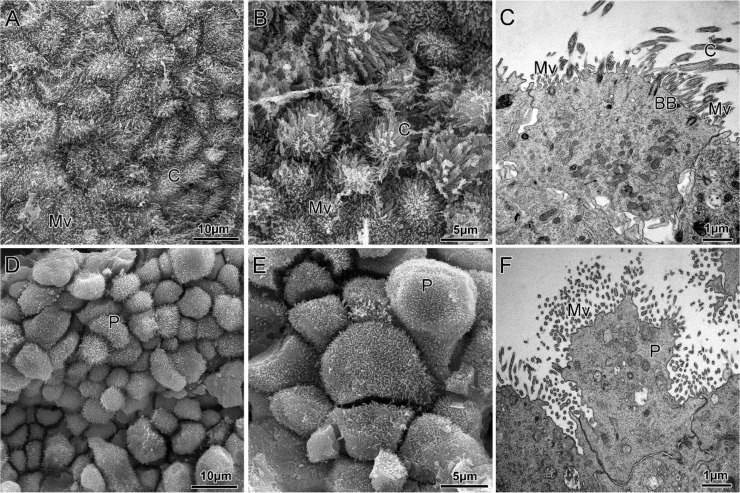
Ultrastructure of the organoid luminal surface showing remarkable changes, the feature of the in vivo endometrium architecture. Representative scanning (**A**,**B**) and transmission electron micrographs (**C**) of ORG-pp treated with E2 to mimic the proliferative phase, showing numerous microvilli and cilia expansion from the apical surface of epithelial cells. Representative scanning (**D**,**E**) and transmission electron micrographs (**F**) of ORG-msp treated with E2, P4, cAMP to mimic the mid-secretory phase, showing numerous pinopodes that bulge from the entire luminal cell surface. These large apical cytoplasmic protrusions are characterized by a significant loss of free surface micro-extensions, including cilia and microvilli. C, cilia; Mv, microvilli; BB: basal body; P: pinopodes.

We also provide a scanning electron microscopic (SEM) inspection of endometrial organoids, definitively demonstrating the dramatic changes occurring at the luminal surface following different hormonal treatments. Hence, in ORG-pp, the luminal surface is relatively smooth or it exhibits irregular cytoplasmic micro-extensions; indeed, it appears overlaid with a rich net of microvilli and cilia of different lengths (Figure 3A,B); TEM analysis of these ciliated cells clearly identified centrioles as basal bodies (Figure 3C), showing the typical microtubular structure.

The luminal surface of endometrial ORG-msp but not ORG-pp is characterized by the presence of large apical cytoplasmic protrusions traditionally called “pinopodes”. Their presence is reported to be a specific marker of the implantation window [32] and is therefore an important and novel feature of hereby described ex vivo endometrial model. Pinopodes bulge out of the entire adluminal cell surface, have regular contours and are characterized by a significant loss of free surface micro-extensions, including cilia and microvilli (Figure 3D,E). Such a significant retraction of these extracellular projections (Figure 3F) is essential to make the excess membrane needed for pinopode formation available. The pinopodes’ cytoplasm is characterized by the presence of a low number of organelles while containing numerous coated vesicles, microfilaments, glycogen aggregates and small mitochondria. Analogously to what is reported in vivo, the pinopodes developed by these in vitro cultured organoids are characterized by the presence of well-defined cell separation, another feature reported to be an essential condition for embryo implantation and invasion [33].

### 3.2. Differential Gene Expression Profile Between Human Endometrial Epithelial Organoids and Stromal Cells Reflects Tissue Heterogeneity

**Figure 4:**
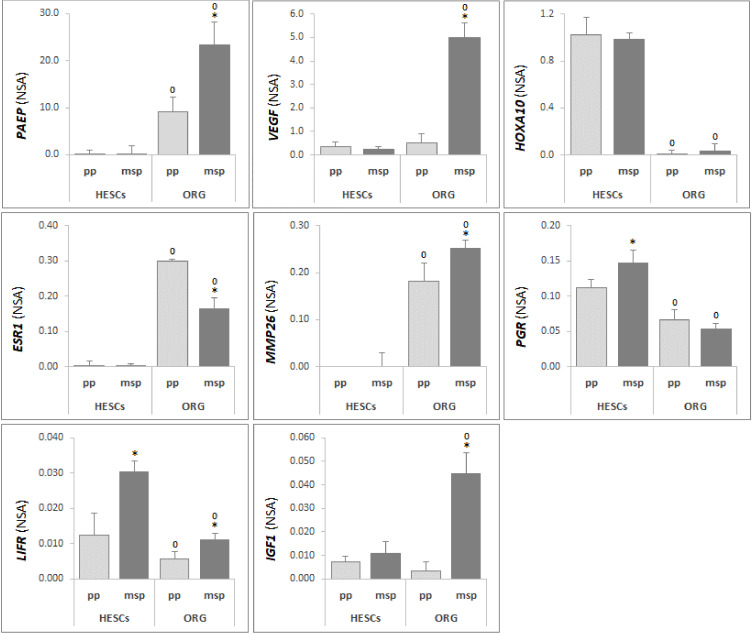
ddPCR analysis of *PAEP*, *VEGF*, *HOXA10*, *ESR1*, *MMP26*, *PGR*, *LIFR* and *IGF1*, from human endometrial stromal cells (HESCs) and from in vitro cultured organoids (ORG), treated in order to mimic the proliferative (pp) and the mid-secretory (msp) phases of the menstrual cycle. Results were expressed in terms of fold change using *GAPDH*, β-*Actin*, *HPRT-1*, *B2M* and *TBP* as the housekeeping genes. Error bars are mean ± S.D., n = 3 independent experiments. Detailed statistical tests are described in the Methods. * msp respect to pp (*p* < 0.05); °ORG respect to HESCs, pp vs pp and msp vs msp (*p* < 0.05).

Endometrial stromal and epithelial cells have separate, yet complementary and inter-related functions in the endometrial physiology. Significant phase-dependent changes in endometrial tissue cellular composition during the menstrual cycle have been reported [34], and this phenomenon may overshadow the real contribution of each cell type to gene expression variations in the whole-tissue samples. In order to compare the gene expression profile of in vitro cultured organoids and endometrial stromal cells (HESCs) obtained from the same patients, we analyzed a panel of genes previously shown to have a differential expression and likely playing a key regulatory role along with the proceedings through the phases of a menstrual cycle. As showed in Figure 4, ddPCR analysis of the two different endometrial cell types revealed significant and dynamic variations in gene expression profile, dependent on different hormonal treatments, inducing either proliferative or secretory phases. The two kinds of cells are markedly different as in regards of the expression of selected genes, namely *PAEP*, *VEGF*, *HOXA10*, *ESR1*, *MMP26*, *PGR*, *LIFR* and *IGF1*.

The expression of *PAEP*, *ESR1* and *MMP26* was significantly higher in epithelial organoids, whereas *PGR* and *LIFR* resulted in increased HESCs if compared to ORG, confirming their key role in decidualization [35,36].

Our results also demonstrated that hormonal treatments are able to affect the expression of genes involved in endometrial receptivity, according to what expected for the different phases of the endometrial cycle; *PAEP*, *VEGF*, *MMP26* and *IGF1*, and appear significantly increased in ORG-msp (Figure 4), thus confirming these molecules as suitable markers of implantation window and proving this in vitro approach as an effective model to closely mimic the molecular modifications typically occurring in vivo during endometrial remodeling.

### 3.3. Human Endometrial Organoids Recapitulate the Glycodelin (GdA) Molecular Signature In Vivo, a Distinguishing Feature of Different Phases of the Uterine Cycle

**Figure 5:**
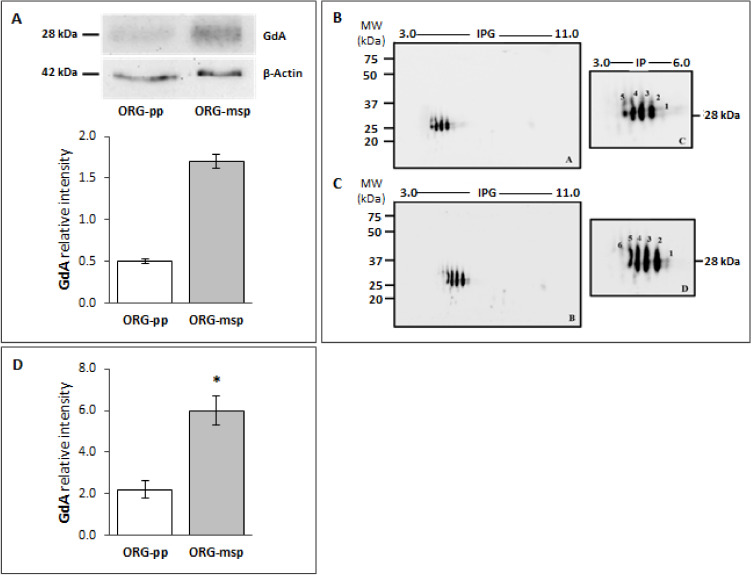
The expression profile of glycodelin-A (GdA) in human endometrial organoids maintains the same signature of the in vivo endometrium. (**A**) Immunoblot analyses with an anti-glycodelin antibody (presumed molecular weight of 28 kDa) of ORG-pp and ORG-msp after mono-dimensional electrophoresis. β-Actin (42 kDa) was immunodetected to control for loading per lane. The computer-assisted semiquantitative analysis of the overall relative intensity of the bands demonstrated a significant increase of GdA expression in organoids mimicking the mid-secretory phase. The intensity was measured (pixel/mm^2^) and then normalized relative to β-Actin. Values are expressed as mean ±SD. The experiments were repeated twice, independently, with similar results. (**B**) Immunoblotting detection of GdA after two-dimensional (2-D) electrophoresis in ORG-pp demonstrates the presence of 4 main isoforms, with a barely detectable fifth one. The experiments were repeated twice, independently, with similar results. (**C**) Immunoblotting detection of GdA after 2-D electrophoresis in ORG-msp showed that up to 6 glycoforms are expressed. The images are representative of n = 2 independent experiments, which gave similar results. (**D**) Computer-assisted semiquantitative analysis of the overall relative intensity of the GdA reactive spots (pixel/mm^2^) normalized with respect to β-Actin. The graph confirmed a significant increase in GdA level in ORG-msp. Values are expressed as mean ± SD. *p < 0.05.

Beside observing an increased *PAEP* mRNA level in ORG-msp, we also focused our attention to the *PAEP*-encoded protein, by GdA reported to exert a key role in endometrial receptivity. Western blot analysis showed that GdA expression could be modulated by different hormonal treatments, with a significant increase of its expression in ORG-msp, mimicking well what observed in vivo in the mid secretory phase (Figure 5A).

Based on the prior knowledge that specific glycoforms of GdA are expressed differentially during the different phases of the menstrual cycle [26], we were curious to see if this fine regulation mechanism is also featured in our 3D model. Therefore, GdA glycoforms pattern was analysed in organoids by two-dimensional electrophoresis. This method highlighted that organoids indeed express different isoforms detected as several spots distributed within a more acidic pH range, with an apparent molecular weight encompassed between 25–37 kDa. In particular, five spots could be detected in ORG-pp, corresponding to five known glycoforms of GdA, with the less acidic spot (spot number 1) being scarcely evident (Figure 5B); instead, in ORG-msp, 6 glycoforms are expressed; among them four glycoforms were prominent, while the forms 1 and 6 (spotted only in this model) were barely identifiable (Figure 5C). Therefore, as summarized in Figure 5D, comparative evaluation of total GdA expression levels, as well as of its gluco-isoforms, in organoids after hormonal treatments is in full agreement with our previously reported data in whole endometrial tissues [26], indicating a peak in GdA expression at the implantation window corresponding to a mid-secretory phase. The appearance of a specific isoform, absent in the proliferative phase model is the additional feature observed in a 2D or 3D model. Current data not only supports the involvement of this molecule in endometrial receptivity but also provided strong evidence for the epithelial cell origin of GdA.

### 3.4. Organoids Isolated from Eutopic Endometrium of Endometriosis-Affected Women, Recapitulate Disease Traits

In order to assess if the epithelial cell 3D-model may also recapitulate endometriosis features, we established the culture from eutopic endometrium of women affected by endometriosis (EndORG). Morphological features and in vitro proliferative rate of EndORG strictly resemble those of organoids prepared from healthy endometrium (Figure S1).

**Figure 6:**
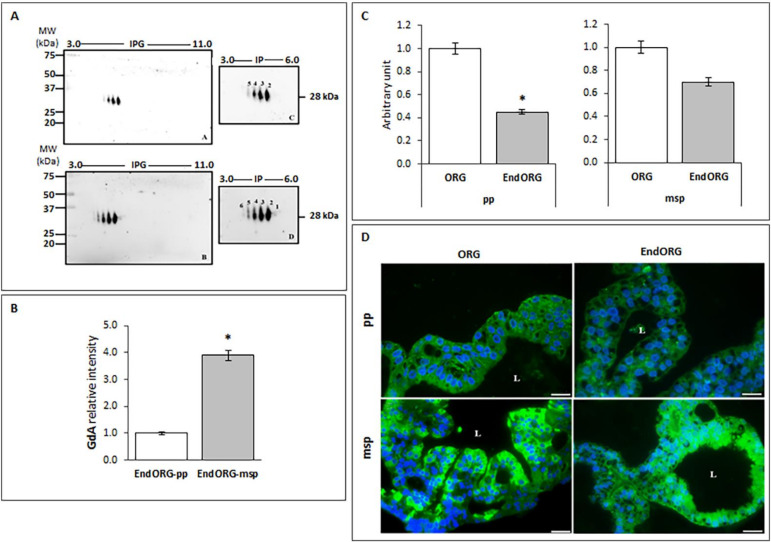
The EndORGGdA signature recapitulates disease traits. (**A**) Immunoblotting detection of GdA after 2-dimensional (2-D) electrophoresis in EndORG-pp (upper panel) that revealed 5 main glycoforms, with spot number 1 scarcely visible. Immunoblotting detection of GdA in EndORG-msp (bottom panel) showed that four glycoforms are prominent, while the spots 1 and 6 are barely identifiable. (**B**) The computer-assisted semiquantitative analysis of the overall relative intensity of the GdA reactive spots (pixel/mm^2^), normalized with respect to β-Actin, demonstrated a significant decrease in EndORG-pp, mimicking the proliferative phase. The experiment was repeated twice, independently, with similar results. (**C**) Computer-assisted semiquantitative analysis of the overall relative intensity of the GdA reactive spots in ORG and EndORG mimicking the proliferative (pp) and the mid secretory (msp) phases of the endometrial cycle. By comparing the GdA quantitative expression between ORG and EndORG, we highlight a significant decrease at the proliferative phase in EndORG. * *p* < 0.05. Values are expressed as mean ± SD. The experiment was repeated twice, independently, with similar results. (**D**) Representative microphotographs of GdA immunofluorescence staining in ORG and EndORG treated to mimic the proliferative (pp) and the mid secretory (msp) phase of the menstrual cycle. GdA, green; 4′,6-diamidino-2-phenylindole (DAPI), blue. The images are representative of *n* = 3 independent experiments, which gave similar results. Scale bar, 50 μm. L: lumen.

Therefore, we evaluated GdA expression pattern in this 3D-disease model: during oestrogen treatment inducing the proliferative phase (EndORG-pp), two-dimensional electrophoresis evidenced four glycoforms, while spots 1 and 6 were not detectable (Figure 6A). By contrast, in organoids treated with oestrogen, progesterone and cAMP (EndORG-msp) all the six glycoforms could be identified, with spots 1 and 6 being clearly visible (Figure 6A). The semi-quantitative analysis of the overall relative intensity of GdA spots (Figure 6B) showed that the EndORG-msp displayed a significantly increased level of GdA (*p* < 0.05) in comparison to EndORG-pp. On the other hand, by comparing GdA quantitative expression in organoids isolated from healthy and eutopic endometrium (Figure 6C), a statistically significant decrease was detected in EndORG-pp with respect to ORG-pp (*p* < 0.05), while no significant difference was observed during mid secretory phase.

The decrease in GdA expression in patients with endometriosis that we reported here by in vitro organoid model confirms data already displayed in endometrial tissue biopsies, further confirming that this model faithfully recapitulates in vivo endometrium [26].

Furthermore, the subcellular localization of GdA in organoids isolated from healthy and eutopic endometrium has been evaluated by immunofluorescence. As showed in Figure 6D, fluorescent staining revealed that GdA is present in all the analyzed sections, but with slight differences. In fact, in both ORG-pp and EndORG-pp, it is possible to observe a clear and diffuse signal, consistent with a cytoplasmic distribution of the protein (Figure 6D). Instead, GdA could be mainly localized in the apical region of the cells in ORG-msp, facing the lumen (Figure 6D), whether it could be detected only in the inner cellular part in EndORG-msp (Figure 6D).

## 4. Discussion

Successful embryo implantation is an essential requirement for reproduction in mammalian species. In humans, this process involves complex interactions between the embryo and the maternal endometrium, all of which must be carried out in an optimal time frame. Endometrial receptivity plays a pivotal role in this process, and consequently, in assisted reproductive techniques, where often high-quality embryos are transferred but the implantation remains the rate-limiting step for the success of treatment [37,38].

Hereby, we validated human endometrial glandular organoids as a powerful 3D-model responding to hormonal treatment by recapitulating in vivo some key morphological, ultrastructural and comprehensive molecular features of the endometrium along with the different phases of the uterine menstrual cycle. The setup of a reliable, reproducible and flexible in vitro model of human endometrium responds to the need for overcoming the ethical issues related to the study of human implantation in vivo. Indeed, in the last years, 3D-culture systems have gained increasing interest due to their evident advantages in providing more physiologically relevant more reliable information [39]. Our study goes beyond the to-date reported establishment and characterization of hormone-responsive endometrial organoids model [9,10,11,12,13]. Beside demonstrating by TEM analysis that this in vitro culture recapitulates well the in vivo glandular architecture according to the different cycle’s phases, SEM analysis gave us the opportunity to provide clear evidence for the presence of pinopodes at the luminal surface of the in the secretory phase-induced organoids. Many reports suggested pinopodes as reliable key features of the implantation window, the limited time frame when endometrium is fully receptive to blastocyst implantation [32]. To the best of our knowledge, this is the first time that this peculiar cellular morphology, strictly related to the topographic localization on the implantation site, is recapitulated in an in vitro model of human endometrium. Therefore, this 3D-model represents physiologically appropriate tool enabling us to study endometrial receptivity in vitro/ex vivo, allowing us to overcome the ethical implications that until now have limited the elucidation of the mechanisms underlying the embryo implantation process and differences due to pathological states or, potentially inter-personal diversity.

We also provided evidence for the phase-dependent presence of cilia on the surface of the luminal epithelium. Conflicting evidence exists today about their presence in the human endometrium. In organoids in the proliferative phase, we highlighted an evident increase in the number of cilia, thus confirming in vitro the oestrogen-regulated endometrial ciliogenesis according to what was already reported [40]. The dramatic decrease in ciliated cells observed in organoids mimicking the mid-secretory phase confirms data from the literature reporting the absence of cilia in epithelium of fertile patients as a positive marker of implantation [41,42]. Indeed, the regular beating of endometrial cilia during the window of implantation may hinder the adhesion of blastocyst to the endometrium, thus decreasing the chances of nidation. This hypothesis is also supported by the presence of ciliated cells in the luminal endometrium of patients with recurrent implantation failure [41].

In our hands, we confirm that functional human endometrial gland organoids respond to hormonal treatment by recapitulating also the molecular signature of the human endometrium observed in vivo. The fine regulation of the expression of selected genes involved in human endometrial receptivity proves the straightness of this model; for instance, ESR1 that has a well-known role in promoting human epithelial cell proliferation [43] as well as *MMP26*, involved in the extracellular matrix remodeling at the implantation window, are found expressed exclusively in endometrial epithelial cells [44].

To-date a large number of genes and markers have been implicated in determining endometrial receptivity and the lack of clear explanation about their roles and interactions made it extremely difficult to display a comprehensive overview of the process, both in particular and in a holistic way. GdA remains considered one of the most valuable candidate markers likely to regulate endometrial receptivity at multi-layer molecular levels [23]. In the present model, not only the overall gene and protein expression levels of GdA are dependent on the hormonal treatment, but also its glycosilation pattern dynamically changes following the expectations based on the results obtained in cycling endometrium in vivo [26]. Interestingly, in a recent study, we demonstrated that both the overall expression and the glycoforms pattern of GdA are altered in eutopic endometrium of women affected by endometriosis [26]. This specific expression pattern is faithfully recapitulated by organoids prepared from eutopic endometrium of endometriotic patients, although their expandability and ultrastructural architecture are matching those prepared from healthy endometrium. Our results are also in agreement with the data from literature reporting an increased GdA level in serum and peritoneal fluid of women affected by endometriosis [45,46], thus supporting the idea that this protein should be considered as a potential biomarker of endometriosis. The significant decrease in GdA expression in eutopic versus healthy organoid mimicking the window of implantation not only confirms data we previously published on endometrial tissue, but is also in agreement with other reported studies [47].

In conclusion, we describe a method for a reliable culture of endometrial epithelial cells that closely recapitulates the molecular and functional characteristics of their cells/tissue of origin. Organoids provide powerful tools for mimicking and examining endometrial development, regulation and disease features, without the presence of the mesenchymal compartment of the tissue. Nevertheless, co-culture with endometrial stromal cells can further implement the organoid model, given the reciprocal epithelium-stroma interactions working in the tissue. Moreover, the development of endometrial organoids also offers the possibility to analyze normal and pathological embryo-maternal interactions and to pin down the key factors involved in the embryo implantation process. Finally, this 3D-model could represent an invaluable research tool to study therapies for common endometrial pathologies, such as endometriosis, as well as investigating implantation problems in natural and assisted reproduction. In conclusion, the possibility to build up a patient-specific biobank resource may be taken into great consideration.

## Supplementary Materials

The following are available online at https://www.mdpi.com/2073-4409/9/5/1121/s1, Table S1; PrimePCR™ Expression Probe Assay, specific for Droplets Digital Polymerase Chain Reaction. Figure S1: Ultrastructure of epithelial glandular organoids in endometrium from patients affected by endometriosis.
