# Supplementary material for: Organoids of Human Endometrium: A Powerful In Vitro Model for the Endometrium-Embryo Cross-Talk at the Implantation Site
Source: Cells. 2020 Apr 30;9(5):1121. doi: 10.3390/cells9051121 (PMC7291023; doi:10.3390/cells9051121)
Supplement: Supplementary file 1 [file cells-09-01121-s001.pdf]

Supplementary Table S1. PrimePCR™ Expression Probe Assay, specific for Droplets Digital Polymerase Chain Reaction.

Gene	Acronym	Catalog code	UniqueAssayID
Target genes			
Leukemia Inhibitory Factor Receptor	*LIFR*	10031252	dHsaCPE5045964
Progestagen-Associated Endometrial Protein	*PAEP*	10031252	dHsaCPE5026616
Estrogen Receptor 1	*ESR1*	10031255	dHsaCPE5033301
Insulin-like Growth Factor 1	*IGF1*	10031255	dHsaCPE5031559
Homeobox A10	*HOXA10*	10031255	dHsaCPE5044351
Forkhead box L2	*FOXL2*	10031255	dHsaCPE5042195
Progesterone Receptor	*PGR*	10031252	dHsaCPE5058418
Vascular Endothelial Growth Factor	*VEGF*	10031255	dHsaCPE5034757
Matrix Metallopeptidase 26	*MMP26*	10031252	dHsaCPE5192861
Reference genes			
Hypoxanthine Phosphoribosyltransferase 1	*HPRT1*	10031255	dHsaCPE5192872
TATA Box Binding Protein	*TBP*	10031255	dHsaCPE5058363
Beta-2-Microglobulin	*B2M*	10031255	dHsaCPE5053101
Actin, Beta	*ACTB*	10031255	dHsaCPE5190200
Glyceraldehyde-3-Phosphate Dehydrogenase	*GAPDH*	10031255	dHsaCPE5031597

Supplementary Figure S1

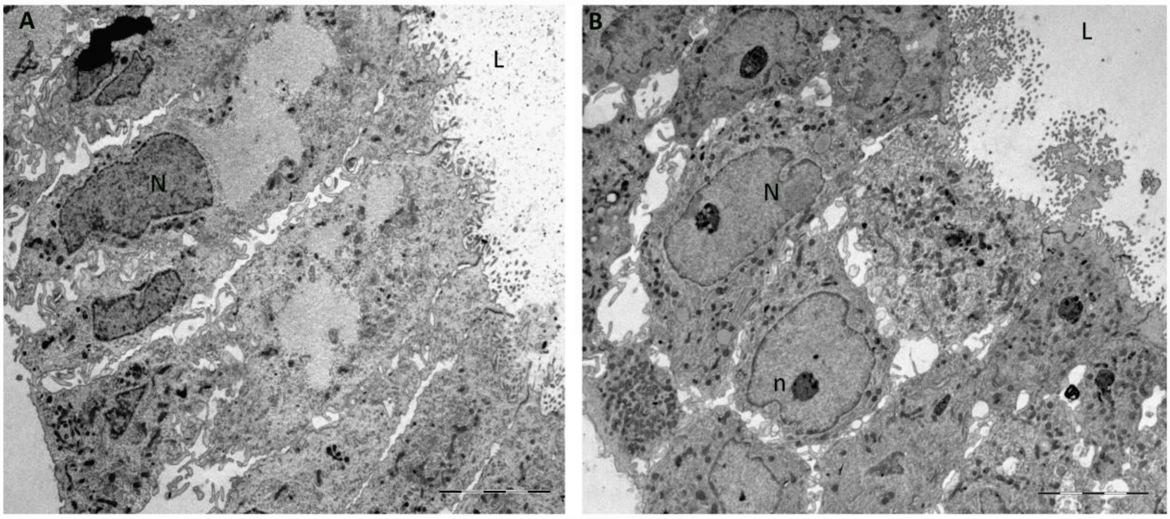

Supplementary Figure S1. Ultrastructure of epithelial glandular organoids recapitulating the physiological architecture of glands in endometrium from patients affected by endometriosis. (A) Representative electron micrograph of ORG-pp treated with E2 to mimic the proliferative phase, showing columnar epithelial cells forming a pseudostratified epithelium. (B). Representative electron micrograph of ORG-msp treated with E2, P4, cAMP to mimic the mid-secretory phase, showing a pluristratified epithelium lining the lumen. L, lumen; N, nucleus; n, nucleolus. Scale bars, 5 µm.
